# Once-weekly IcoSema versus once-weekly semaglutide in adults with type 2 diabetes: the COMBINE 2 randomised clinical trial

**DOI:** 10.1007/s00125-024-06348-5

**Published:** 2025-01-17

**Authors:** Ildiko Lingvay, Malik Benamar, Liming Chen, Ariel Fu, Esteban Jódar, Tomoyuki Nishida, Jean-Pierre Riveline, Daisuke Yabe, Thomas Zueger, Rosângela Réa

**Affiliations:** 1 Endocrinology Division, Department of Internal Medicine and Peter O’Donnell Jr School of Public Health, University of Texas Southwestern Medical Center, Dallas, TX USA; 2 Novo Nordisk A/S, Søborg, Denmark; 3 NHC Key Laboratory of Hormones and Development, Tianjin Key Laboratory of Metabolic Diseases, Chu Hsien-I Memorial Hospital and Tianjin Institute of Endocrinology, Tianjin Medical University, Tianjin, China; 4 Universitary Hospital QuironSalud Madrid, Faculty of Biomedical and Health Sciences, Universidad Europea de Madrid, Madrid, Spain; 5 Novo Nordisk Pharma Ltd, Tokyo, Japan; 6 Department of Endocrinology and Diabetology, Lariboisière Hospital, APHP, Paris-Cité University Paris, Paris, France; 7 Institut Necker Enfants Malades, Inserm U1151, CNRS UMR 8253, IMMEDIAB Laboratory, Paris, France; 8 Department of Diabetes, Endocrinology and Nutrition, Kyoto University Graduate School of Medicine, Kyoto, Japan; 9 Department of Endocrinology, Diabetes and Metabolic Diseases, Kantonsspital Olten, Olten, Switzerland; 10 Department of Diabetes, Endocrinology, Nutritional Medicine and Metabolism, Inselspital, Bern University Hospital, University of Bern, Bern, Switzerland; 11 Internal Medicine Department, Endocrine Division (SEMPR), Universidade Federal do Paraná, Curitiba, Brazil

**Keywords:** Fixed-ratio combination, GLP-1 RA, Glycaemic control, Hypoglycaemia, IcoSema, Insulin icodec, Once-weekly, Safety, Semaglutide, Type 2 diabetes

## Abstract

**Aims/hypothesis:**

COMBINE 2 assessed the efficacy and safety of once-weekly IcoSema (a combination therapy of basal insulin icodec and semaglutide) vs once-weekly semaglutide (a glucagon-like peptide-1 analogue) 1.0 mg in individuals with type 2 diabetes inadequately managed with GLP-1 receptor agonist (GLP-1 RA) therapy, with or without additional oral glucose-lowering medications.

**Methods:**

This 52 week, randomised, multicentre, open-label, parallel group, Phase IIIa trial was conducted across 121 sites in 13 countries/regions. Adults with type 2 diabetes (HbA_1c_ 53.0–85.8 mmol/mol [7.0–10.0%]) receiving GLP-1 RA therapy with or without additional oral glucose-lowering medications were randomly assigned 1:1 to once-weekly IcoSema or once-weekly semaglutide 1.0 mg. The primary endpoint was change in HbA_1c_ from baseline to week 52; superiority of IcoSema to semaglutide 1.0 mg was assessed. Secondary endpoints included change in fasting plasma glucose and body weight (baseline to week 52), and combined clinically significant (level 2; <3.0 mmol/l) or severe (level 3; associated with severe cognitive impairment requiring external assistance for recovery) hypoglycaemia (baseline to week 57).

**Results:**

Overall, 683 participants were randomised using a Randomisation and Trial Supply Management system to IcoSema (*n*=342) or semaglutide 1.0 mg (*n*=341). Mean ± SD baseline characteristics were as follows: HbA_1c_ 64.0±8.2 mmol/mol (8.0±0.7%); diabetes duration 12.6±6.9 years; and BMI 31.1±4.7 kg/m^2^. From baseline to week 52, mean change in HbA_1c_ was −14.7 mmol/mol (−1.35%-points) in the IcoSema group and −9.88 mmol/mol (−0.90%-points) in the semaglutide group; the estimated treatment difference (ETD) was –4.85 (95% CI −6.13, −3.57) mmol/mol (−0.44 [95% CI −0.56, −0.33]%-points), confirming superiority of IcoSema to semaglutide (*p*<0.0001). The estimated mean change in fasting plasma glucose from baseline to week 52 was statistically significantly reduced with IcoSema vs semaglutide (−2.48 mmol/l vs −1.41 mmol/l, respectively; ETD −1.07 [95% CI −1.37, −0.76] mmol; *p*<0.0001). Mean change in body weight from baseline to week 52 was statistically significantly different between groups: +0.84 kg for IcoSema vs −3.70 kg for semaglutide (ETD 4.54 kg [95% CI 3.84, 5.23]; *p*<0.0001). There was no statistically significant difference in the rate of combined clinically significant or severe hypoglycaemia between IcoSema and semaglutide (0.042 vs 0.036 episodes per person-year of exposure; estimated rate ratio 1.20 [95% CI 0.53, 2.69]; *p*=0.66). The proportion of participants experiencing gastrointestinal adverse events was similar between treatment groups (IcoSema 31.4%; semaglutide 34.4%).

**Conclusions/interpretation:**

In people living with type 2 diabetes inadequately managed with GLP-1 RA therapy, with or without additional oral glucose-lowering medications, switching to once-weekly IcoSema in comparison with once-weekly semaglutide 1.0 mg demonstrated superiority in HbA_1c_ reduction, similar rates of clinically significant or severe hypoglycaemia, and similar frequency of gastrointestinal adverse events. However, weight change from baseline to week 52 was statistically significantly in favour of semaglutide 1.0 mg.

**Trial registration:**

ClinicalTrials.gov NCT05259033

**Funding:**

This trial was funded by Novo Nordisk

**Graphical Abstract:**

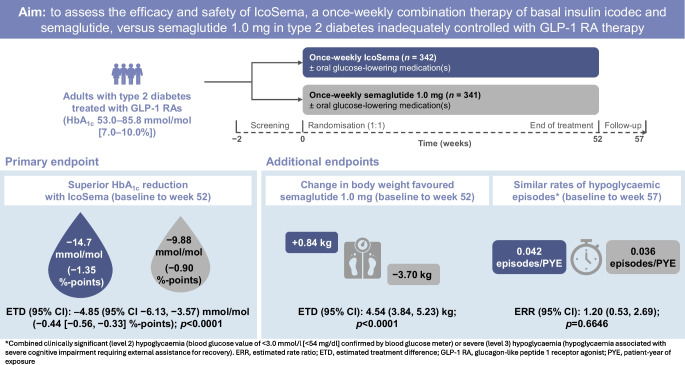

**Supplementary Information:**

The online version contains peer-reviewed but unedited supplementary material available at 10.1007/s00125-024-06348-5.


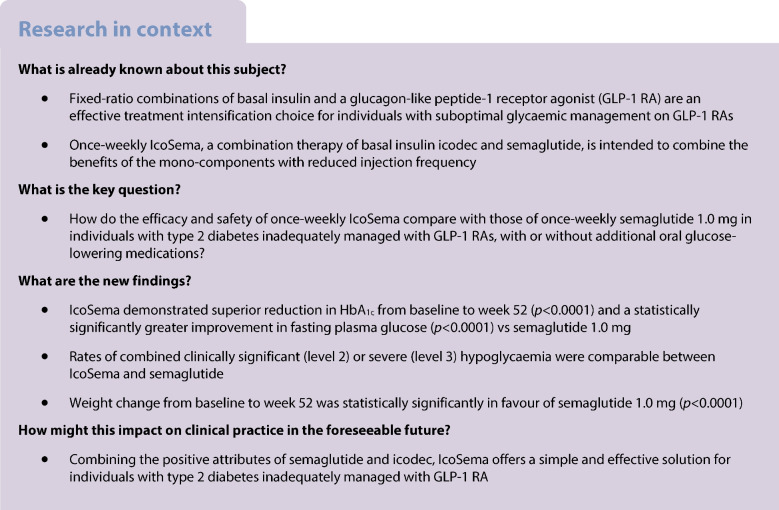


## Introduction

Owing to the progressive nature of type 2 diabetes, and despite the availability of drugs that provide benefits beyond the reduction of blood glucose levels, disease progression is hallmarked by relative insulin deficiency. Consequently, many people need treatment with insulin to achieve and maintain glycaemic control. When diabetes progresses to the point that several non-insulin glucose-lowering medications are no longer effective in providing individuals with adequate glucose management, the initiation of insulin is recommended [1, 2].

The use of fixed-ratio combinations of basal insulin and a glucagon-like peptide-1 receptor agonist (GLP-1 RA), such as the once-daily formulations insulin degludec/liraglutide (IDegLira) or insulin glargine 100 U/ml plus lixisenatide (IGlarLixi), has been shown to be efficacious with respect to improving glycaemic management while also being associated with other benefits such as less weight gain, a lower risk of hypoglycaemia and a lower insulin dose than with basal bolus insulin therapy.

IcoSema, a once-weekly combination therapy of basal insulin icodec (hereafter referred to as icodec) and semaglutide, is intended to combine the known benefits of the mono-components with a reduced injection frequency. One dose step of IcoSema is equivalent to 1 U icodec and 0.0029 mg semaglutide. The maximum weekly dose of IcoSema is 350 dose steps, corresponding to 350 U of icodec (50 U of basal insulin per day) and 1.0 mg of semaglutide. Icodec is a basal insulin analogue studied in six phase 3a randomised trials as part of the ONWARDS clinical trial programme in individuals with type 2 diabetes or type 1 diabetes [3–8]. The efficacy and safety of injectable semaglutide 1.0 mg, a GLP-1 RA, were established by the SUSTAIN clinical trial programme in individuals with type 2 diabetes, along with real-world studies [9–16].

IcoSema is intended for the management of type 2 diabetes that is insufficiently controlled on basal insulin or GLP-1 RAs in addition to diet, exercise and oral glucose-lowering medications. Once-weekly IcoSema was evaluated in the phase 3a COMBINE programme, comprising three 52 week, multinational, multicentre, randomised, open-label trials in individuals with type 2 diabetes (COMBINE 1–3). COMBINE 2 was the first assessment of the efficacy and safety of once-weekly IcoSema vs once-weekly semaglutide 1.0 mg in individuals with type 2 diabetes inadequately managed with GLP-1 RA therapy, with or without additional oral glucose-lowering medications.

## Methods

### Trial design and participants

COMBINE 2 (ClinicalTrials.gov registration no. NCT05259033) was a 52 week, randomised, multicentre, open-label, parallel group, phase 3a trial. It was conducted across 121 sites in 13 countries/regions (Brazil, Canada, mainland China, France, Greece, Hungary, Israel, Japan, Slovakia, Sweden, Switzerland, Taiwan and the USA; see the list of primary investigators and trial sites in the electronic supplementary material [ESM]). The trial consisted of a 2 week screening period, a 52 week treatment period and a 5 week follow-up period (ESM Fig. 1). Eligible participants were adults (≥18 years old) with inadequately managed type 2 diabetes (HbA_1c_ 53.0–85.8 mmol/mol [7.0–10.0%]), treated with stable doses of daily or weekly GLP-1 RAs (excluding once-weekly semaglutide with doses higher than 1.0 mg), according to the local label, for the treatment of type 2 diabetes for at least 90 days before screening, with or without stable doses of additional oral glucose-lowering medications. Full inclusion and exclusion criteria are provided in ESM Table 1 and representativeness of the study participants is detailed in ESM Table 2.

### Trial treatment

A Randomisation and Trial Supply Management system was used to randomly assign participants (1:1) to IcoSema or semaglutide 1.0 mg once weekly. Participants continued treatment with pre-trial non-insulin glucose-lowering medications except for GLP-1 RA, sulfonylurea, glinide and dipeptidyl peptidase-4 inhibitor (DPP-4i) therapies, which were discontinued at randomisation.

IcoSema was administered once weekly on the same day each week at any time of day. The starting dose of IcoSema was 40 dose steps (equivalent to 40 U of icodec and 0.114 mg of semaglutide). Semaglutide was administered once weekly on the same day each week, at any time of the day, irrespective of meals. The starting dose of semaglutide was 0.25 mg. The first dose of IcoSema or semaglutide was administered at least 5 days after the last dose of pre-trial weekly GLP-1 RA or the following day after the last dose of pre-trial daily GLP-1 RA.

IcoSema doses were adjusted in ten dose-step increments or decrements once weekly following a treat-to-target approach based on three pre-breakfast self-measured blood glucose (SMBG) values, measured on the 2 days before titration and on the day of titration (target SMBG: 4.4–7.2 mmol/l) (ESM Table 3). If at least one pre-breakfast SMBG value was missing, the dose adjustment was based on the remaining SMBG value(s).

Semaglutide followed a dose escalation scheme. After 4 weeks of treatment with semaglutide 0.25 mg, the dose was increased to 0.5 mg. After another 4 weeks, the dose was increased to 1.0 mg and maintained at this dose thereafter. If adverse events (AEs) occurred, the escalation to 1.0 mg could be extended up to 26 weeks after randomisation. Dose reductions from 1.0 mg to 0.5 mg were allowed at any point, if there were safety concerns or unacceptable intolerability.

Participants were instructed to take their SMBG reading once daily before breakfast at a minimum but were encouraged to do this as many times as they deemed necessary. Participants were also instructed to record their SMBG value whenever they experienced symptoms of hypoglycaemia or hyperglycaemia.

### Outcomes

The primary endpoint was change in HbA_1c_ from baseline to week 52. Supportive secondary endpoints were: change in body weight from baseline to week 52; change in fasting plasma glucose (FPG) from baseline to week 52; combined clinically significant (level 2; <3.0 mmol/l [<54 mg/dl], confirmed by blood glucose meter) or severe (level 3; associated with severe cognitive impairment requiring external assistance for recovery) hypoglycaemia from baseline to week 57; clinically significant hypoglycaemic episodes from baseline to week 57; and severe hypoglycaemic episodes from baseline to week 57.

Additional assessments were: pre-breakfast SMBG by week; treatment dose at weeks 50–52; achievement of HbA_1c_ <53.0 mmol/mol (<7.0%) at week 52; achievement of HbA_1c_ <53.0 mmol/mol (<7.0%) at week 52 without clinically significant or severe hypoglycaemia in the previous 12 weeks; achievement of HbA_1c_ <53.0 mmol/mol (<7.0%) at week 52 without weight gain and without clinically significant or severe hypoglycaemia in the previous 12 weeks; and changes from baseline in BP, waist circumference and lipids. Investigators were responsible for reporting AEs throughout the trial.

### Statistical analysis

All statistical analyses reported here were pre-specified. Efficacy endpoints were evaluated using the full analysis set (all randomised participants) and data from randomisation until the occurrence of any one of the following: last site contact or investigator contact; withdrawal; death; or end-of-trial visit. Safety endpoints were evaluated using the period in which a participant was exposed to the trial product; descriptive statistics were evaluated using the safety analysis set (participants exposed to the trial product) and statistical analyses were based on the full analysis set.

The primary hypothesis was that IcoSema is superior to semaglutide 1.0 mg treatment in terms of change in HbA_1c_ from baseline to week 52. The primary estimand evaluated the treatment effect of the primary endpoint for all randomised participants, irrespective of adherence to trial product, initiation of non-randomised insulin treatment or addition of other non-insulin glucose-lowering medications (for more than 2 weeks) (i.e. intercurrent events [initiation of non-randomised insulin treatment or addition of other non-insulin glucose-lowering medications and discontinuation of randomised treatment] were handled by the treatment policy strategy). The secondary estimand definitions are provided in ESM Table 4. Based on previous studies with IDegLira and semaglutide, a sample size of 680 participants was determined to have at least 90% power for superiority of IcoSema to semaglutide assuming a treatment difference of −2.99 mmol/mol (−0.274%-points) change in HbA_1c_ and an SD of 12.02 mmol/mol (1.1%-points).

The primary endpoint, changes in body weight and changes in FPG were analysed using an ANCOVA model with region and randomised treatment as fixed factors and the baseline value as a covariate. Hypoglycaemia endpoints were analysed using a negative binomial model with log-link function, including treatment, region and the logarithm of the time period in which a participant was exposed to the trial product as offset. Achievement of target HbA_1c_ <53.0 mmol/mol (<7.0%) was analysed using a logistic regression model with region and randomised treatment as fixed factors, and baseline HbA_1c_ value as a covariate. Weekly treatment dose and AEs were reported descriptively. Missing data were imputed using multiple imputation for all endpoints analysed (further details provided in ESM Methods).

### Trial oversight

This trial was conducted in compliance with the principles of the Declaration of Helsinki and in accordance with the Good Clinical Practice guidelines of the International Council for Harmonisation. The protocol, consent form and other relevant documents were reviewed and approved by the appropriate institutional review boards or independent ethics committees. All participants provided written informed consent before trial entry and could withdraw their consent at any time.

## Results

### Participant disposition

**Fig. 1:**
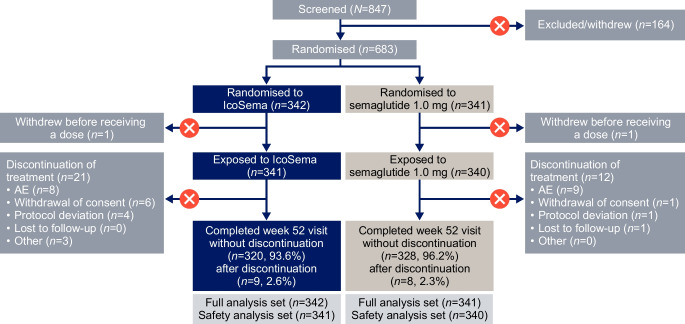
Participant disposition flow diagram

Participants were recruited between 11 April 2022 and 30 November 2022. Of 847 screened participants, 683 were randomised to IcoSema (*n*=342) or semaglutide 1.0 mg (*n*=341) (Fig. 1). One participant in each group withdrew before receiving any trial product. All randomised participants were included in the full analysis set, and the 681 participants (IcoSema, *n*=341; semaglutide, *n*=340) who received at least one dose of trial product were included in the safety analysis set. In the IcoSema group, 320 participants (93.6%) completed the week 52 visit without permanent discontinuation of the trial product. In the semaglutide group, 328 participants (96.2%) completed the week 52 visit without permanent discontinuation of trial product. The most common reason for discontinuation of trial product in both treatment groups was AEs (Fig. 1).

Baseline demographics and clinical characteristics were comparable between treatment groups (Table 1). In the IcoSema and semaglutide groups, respectively, mean ± SD HbA_1c_ was 64.7±8.3 mmol/mol (8.07±0.76%) and 63.2±8.0 mmol/mol (7.93±0.73%) and mean ± SD diabetes duration was 12.94±6.82 years and 12.34±6.97 years. Body weight at baseline was slightly higher in the semaglutide group than in the IcoSema group (90.8 kg vs 87.6 kg, respectively). A similar pattern was observed for BMI, and the mean BMI at baseline would be classified as class I obesity in both groups [17].

**Table 1:** Participant demographics and baseline characteristics

Characteristic	IcoSema (*n*=342)	Semaglutide 1.0 mg (*n*=341)	Total (*n*=683)
Sex, male	201 (58.8)	196 (57.5)	397 (58.1)
Age, years	59.9±10.5	58.3±9.8	59.1±10.2
Race^a^			
Native American or Alaskan Native	1 (0.3)	0	1 (0.1)
Asian	99 (28.9)	90 (26.4)	189 (27.7)
Black or African American	12 (3.5)	9 (2.6)	21 (3.1)
Native Hawaiian or other Pacific Islander	0	1 (0.3)	1 (0.1)
White	210 (61.4)	224 (65.7)	434 (63.5)
Other^b^	0	2 (0.6)	2 (0.3)
Not reported	20 (5.8)	15 (4.4)	35 (5.1)
Ethnicity^b^			
Hispanic or Latino	37 (10.8)	37 (10.9)	74 (10.8)
Not Hispanic or Latino	285 (83.3)	289 (84.8)	574 (84.0)
Not reported	20 (5.8)	15 (4.4)	35 (5.1)
HbA_1c_, mmol/mol	64.7±8.3	63.2±8.0	64.0±8.2
HbA_1c_, %	8.07±0.76	7.93±0.73	8.00±0.75
FPG, mmol/l	9.56±2.71	9.35±2.66	9.45±2.69
Diabetes duration, years	12.94±6.82	12.34±6.97	12.64±6.89
Body weight, kg	87.58±18.20	90.82±17.74	89.20±18.03
BMI, kg/m^2^	30.58±4.69	31.65±4.66	31.11±4.70
Non-insulin glucose-lowering medications at screening	325 (95.0)	328 (96.2)	653 (95.6)
Metformin	298 (87.1)	305 (89.4)	603 (88.3)
SGLT2i	139 (40.6)	160 (46.9)	299 (43.8)
Sulfonylurea	99 (28.9)	96 (28.2)	195 (28.6)
Thiazolidinedione	22 (6.4)	19 (5.6)	41 (6.0)
α-Glucosidase inhibitor	21 (6.1)	10 (2.9)	31 (4.5)
DPP-4i	15 (4.4)	14 (4.1)	29 (4.2)
Glinide	13 (3.8)	19 (5.6)	32 (4.7)
GLP-1 RAs	339 (99.1)	341 (100.0)	680 (99.6)
Semaglutide^c^	147 (43.0)	173 (50.7)	320 (46.9)
Semaglutide 0.25–1.0 mg	123 (36.0)	146 (42.8)	269 (39.4)
Semaglutide 3–14 mg	24 (7.0)	27 (7.9)	51 (7.5)
Dulaglutide	137 (40.1)	112 (32.8)	249 (36.5)
Liraglutide	49 (14.3)	53 (15.5)	102 (14.9)
Exenatide	4 (1.2)	1 (0.3)	5 (0.7)
Polyethylene glycol loxenatide	2 (0.6)	2 (0.6)	4 (0.6)

Data are presented as mean ±SD or *n* (%)

All participants self-identified their race and ethnicity

^a^Participants from France did not report race or ethnicity and are included as ‘Not reported’

^b^Two participants reporting ‘Other’ for race self-identified as ‘Hawaiian, Chinese, Filipino, Spanish’ and ‘African/South Asian’

^c^Semaglutide subcutaneous or oral

SGLT2i, sodium–glucose cotransporter 2 inhibitor

The most common oral glucose-lowering medications at screening were metformin, sodium–glucose cotransporter 2 inhibitors and sulfonylureas. The most common GLP-1 RAs were semaglutide (including subcutaneous and oral semaglutide) and dulaglutide (Table 1). Of those participants receiving semaglutide at screening, 20.2% of participants in the IcoSema group and 24.3% of participants in the semaglutide group were receiving semaglutide 1.0 mg.

Changes to background glucose-lowering medications that occurred after randomisation and lasted more than 2 weeks are summarised in ESM Table 5. From baseline until 1 week after the last dose of randomised treatment, a total of 13 participants (3.8%) in the IcoSema group and 71 participants (20.8%) in the semaglutide group initiated non-randomised insulin or additional non-insulin glucose-lowering medications for more than 2 weeks, with most of them initiating oral glucose-lowering medications rather than insulin treatment.

### Primary outcome

**Fig. 2:**
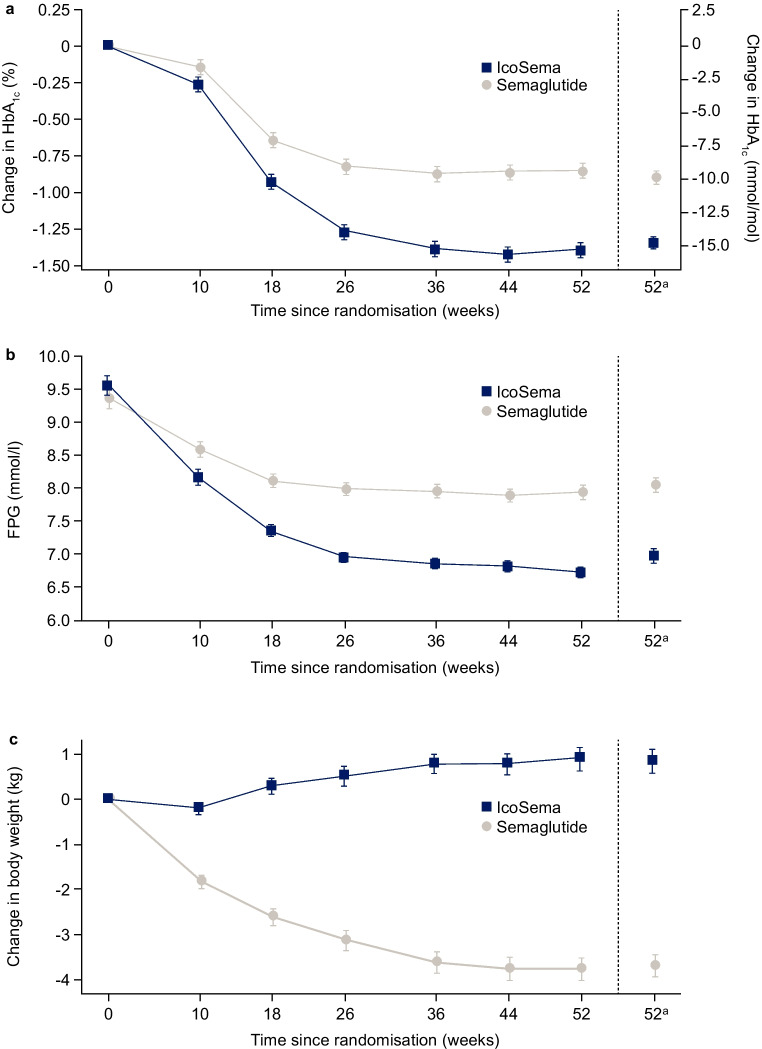
Observed key efficacy and safety outcomes in the full analysis set (IcoSema, *n*=342; semaglutide, *n*=341). (**a**) Change in HbA_1c_ from baseline, (**b**) change in FPG over time and (**c**) change in body weight from baseline. Data are presented as mean ± SEM. ^a^The estimated mean values and the corresponding SEM at week 52 were derived based on an ANCOVA model on multiple imputed data

In the IcoSema group, mean HbA_1c_ decreased from 64.7 mmol/mol (8.07%; observed) at baseline to 49.2 mmol/mol (6.65%; estimated) at week 52 (estimated change ± SEM −14.7±0.47 mmol/mol [−1.35±0.04%-points]). In the semaglutide group, mean HbA_1c_ decreased from 63.2 mmol/mol (7.93%; observed) at baseline to 54.1 mmol/mol (7.10%; estimated) at week 52 (estimated change ± SEM −9.88±0.45 mmol/mol [−0.90±0.04%-points]). The estimated treatment difference (ETD) was −4.85 (95% CI −6.13, −3.57) mmol/mol (−0.44 [95% CI −0.56, −0.33]%-points), confirming the superiority of IcoSema over semaglutide (*p*<0.0001) (Table 2, Fig. 2a).

**Table 2:** Summary of key efficacy and safety endpoints

Endpoint	LS mean		Treatment difference		
	IcoSema	Semaglutide 1.0 mg	Estimate	95% CI	*p* value
Estimated mean change in HbA_1c_ from baseline to week 52, mmol/mol	−14.7	−9.88	−4.85	−6.13, −3.57	<0.0001^a^
Estimated mean change in HbA_1c_ from baseline to week 52, %-points	−1.35	−0.90	−0.44	−0.56, −0.33	<0.0001^a^
Estimated mean change in FPG from baseline to week 52, mmol/l	−2.48	−1.41	−1.07	−1.37, −0.76	<0.0001
Estimated mean change in body weight from baseline to week 52, kg	0.84	−3.70	4.54	3.84, 5.23	<0.0001

^a^Primary endpoint. Superiority was confirmed based on the upper bound of the two-sided 95% CI treatment difference being strictly <0. Two-sided *p* values are presented. The change from baseline to week 52 in HbA_1c_, FPG and body weight was separately analysed using an ANCOVA model with region and randomised treatment as fixed factors and a baseline value as a covariate

LS, least-squares

### Secondary efficacy outcomes

The estimated mean ± SEM change in FPG from baseline to week 52 was −2.48±0.11 mmol/l in the IcoSema group and −1.41±0.11 mmol/l in the semaglutide group; the mean treatment difference was statistically significantly greater with IcoSema vs semaglutide (ETD −1.07 [95% CI −1.37, −0.76] mmol/l; *p*<0.0001) (Table 2, Fig. 2b).

Estimated mean change in body weight from baseline to week 52 was +0.84 kg with IcoSema and −3.70 kg with semaglutide (ETD 4.54 [95% CI 3.84, 5.23] kg), which was statistically significantly in favour of semaglutide over IcoSema (*p*<0.0001) (Table 2, Fig. 2c).

### Additional assessments

During the treatment period, an improvement in pre-breakfast SMBG values was observed in both groups. Pre-breakfast SMBG showed similar, minor transient increases in the first weeks after randomisation in both treatment groups (ESM Fig. 2). In the initial 3 weeks, the observed mean pre-breakfast SMBG levels increased by 0.31 mmol/l from baseline in the IcoSema group and by 0.47 mmol/l in the semaglutide group but had returned to the baseline level by approximately week 6 in both treatment groups. The mean pre-breakfast SMBG value continued to decline in both groups throughout the trial. In the IcoSema group, the mean SMBG reached and remained within the predefined targets of 4.4–7.2 mmol/l after approximately week 20.

The mean treatment doses during the last 2 weeks of treatment were 195.7 dose steps in the IcoSema group (equivalent to a mean weekly basal insulin component of 195.7 U and a mean weekly semaglutide component of 0.56 mg). At week 52, 97.4% of participants in the semaglutide group had reached the 1.0 mg dose per protocol and 10.8% of participants in the IcoSema group had titrated up to the maximum dose of 350 dose steps (icodec 350 U/semaglutide 1.0 mg).

The odds of achieving HbA_1c_ <53 mmol/mol (<7.0%) at week 52 were statistically significantly higher in the IcoSema group than in the semaglutide group (estimated mean proportion of participants, 73.5% vs 48.0%, respectively; estimated OR [EOR] 3.01 [95% CI 2.14, 4.24]; *p*<0.0001). The odds of achieving HbA_1c_ <53 mmol/mol (<7.0%) at week 52 without clinically significant or severe hypoglycaemia during the previous 12 weeks were statistically significantly greater with IcoSema than with semaglutide (estimated percentage 71.4% vs 45.8%, respectively; EOR 2.95 [95% CI 2.11, 4.12]; *p*<0.0001). In contrast, the odds of achieving HbA_1c_ <53 mmol/mol (<7.0%) at week 52 without weight gain and clinically significant or severe hypoglycaemia during the previous 12 weeks were statistically significantly greater with semaglutide than IcoSema treatment (estimated percentage 40.5% vs 30.2%, respectively; EOR 0.64 [95% CI 0.46, 0.88]; *p*=0.007) (Table 3).

**Table 3:** Summary of composite endpoints

Endpoint	Percentage of participants achieving composite endpoint		OR	95% CI	*p* value
	IcoSema (*n*=342)	Semaglutide (*n*=341)			
Participants achieving HbA_1c_ <53 mmol/mol (<7.0%) at week 52	73.5	48.0	3.01	2.14, 4.24	<0.0001
Participants achieving HbA_1c_ <53 mmol/mol (<7.0%) at week 52 without clinically significant or severe hypoglycaemia during the previous 12 weeks	71.4	45.8	2.95	2.11, 4.12	<0.0001
Participants achieving HbA _1c_ <53 mmol/mol (<7.0%) at week 52 without body weight gain and clinically significant or severe hypoglycaemia in the previous 12 weeks	30.2	40.5	0.64	0.46, 0.88	0.007

Two-sided *p* values are presented. The binary response was separately analysed using a logistic regression model (logit link) with region and randomised treatment as fixed factors, and baseline HbA_1c_ value as a covariate

Changes from baseline to week 52 in BP, waist circumference and lipids are shown in ESM Table 6.

### Safety outcomes

Hypoglycaemia data are presented in Table 4. There were no episodes of severe hypoglycaemia in either treatment group. Similar proportions of participants experienced clinically significant hypoglycaemia in each group (IcoSema 3.5%; semaglutide 3.8%). There was no statistically significant difference in the rate of combined clinically significant or severe hypoglycaemia between IcoSema and semaglutide (observed rate 0.042 vs 0.036 episodes per person-year of exposure [PYE], respectively; estimated rate ratio [95% CI] 1.20 [95% CI 0.53, 2.69]; *p*=0.6646).

**Table 4:** Hypoglycaemia and other AEs

AE	IcoSema (*n*=341)				Semaglutide 1.0 mg (*n*=340)				IcoSema vs semaglutide 1.0 mg, ERR (95% CI)
	*n*	%	E	R	*n*	%	E	R	
Hypoglycaemia (baseline to week 57)									
Hypoglycaemia alert value^a^	68	19.9	214	0.596	21	6.2	52	0.143	
Clinically significant hypoglycaemia^b^	12	3.5	15	0.042	13	3.8	13	0.036	1.20 (0.53, 2.69); *p*=0.66
Severe hypoglycaemia^c^	0				0				
Clinically significant or severe hypoglycaemia	12	3.5	15	0.042	13	3.8	13	0.036	1.20 (0.53, 2.69); *p*=0.66
Summary of AEs (baseline to week 57)									
AEs	270	79.2	1147	3.19	252	74.1	1185	3.25	
Serious AEs	38	11.1	47	0.131	21	6.2	34	0.093	
Most frequently reported (≥5%) AEs									
Gastrointestinal disorders									
Nausea	40	11.7	65	0.181	39	11.5	74	0.203	
Diarrhoea	38	11.1	53	0.148	42	12.4	83	0.228	
Vomiting	18	5.3	25	0.070	22	6.5	49	0.134	
Constipation	11	3.2	14	0.039	17	5.0	26	0.071	
Infections and infestations									
COVID-19	51	15.0	52	0.145	49	14.4	53	0.145	
Nasopharyngitis	33	9.7	45	0.125	33	9.7	45	0.123	
Upper respiratory-tract infection	22	6.5	34	0.095	29	8.5	34	0.093	
Nervous system disorders									
Headache	20	5.9	25	0.070	6	1.8	6	0.016	
Eye disorders									
Diabetic retinopathy	19	5.6	20	0.056	10	2.9	10	0.027	

Safety analysis set. Hypoglycaemia endpoints were analysed using a negative binomial model with log-link function, including region and randomised treatment as fixed factors and the logarithm of the time period in which a participant was exposed to the trial product as offset. ^a^Hypoglycaemia alert value: blood glucose value of <3.9 mmol/l (<70 mg/dl) but ≥3.0 mmol/l (≥54 mg/dl) confirmed by blood glucose meter

^b^Clinically significant hypoglycaemia: blood glucose value of <3.0 mmol/l (<54 mg/dl) confirmed by blood glucose meter

^c^Severe hypoglycaemia: hypoglycaemia with severe cognitive impairment requiring external assistance for recovery

Two-sided *p* values are presented

%, proportion of participants with at least one event; E. number of events; ERR, estimated rate ratio; *n*, no. of participants with one or more events; R, rate (no. of AEs per PYE [1 PYE = 365.25 days])

AEs were reported by 270 participants (79.2%) receiving IcoSema and 252 (74.1%) receiving semaglutide. Most AEs were non-serious, mild or moderate in severity, unlikely to be related to trial products (as judged by the investigator) and events were recovered or recovering by the end of the trial.

Overall, 47 serious AEs were reported by 11.1% of participants in the IcoSema group and 34 events were reported by 6.2% of participants in the semaglutide group. Events were distributed across multiple system organ classes (SOCs) with no observed clustering in either treatment group. Two events in two participants in the IcoSema group and four events in two participants in the semaglutide group were possibly or probably related to the trial product. Two fatal events were reported in the IcoSema group (sudden death and cerebrovascular accident), both determined as unlikely to be related to the trial product by investigator. No fatal events were reported in the semaglutide group.

In both groups, the most frequently reported AEs by SOC were gastrointestinal disorders (preferred terms [PTs]: nausea, diarrhoea, vomiting and constipation) and infections and infestations (PTs: COVID-19, nasopharyngitis and upper respiratory-tract infection) (Table 4). Overall, 2.3% of participants in the IcoSema group and 2.6% of participants in the semaglutide group experienced AEs leading to premature discontinuation of the trial product.

The proportions of participants experiencing gastrointestinal AEs were similar between treatment groups (IcoSema 31.4%; semaglutide 34.4%), with corresponding event rates of 0.67 events/PYE in the IcoSema group vs 0.89 events/PYE in the semaglutide group. Most of the gastrointestinal AEs were non-serious and mild in severity; three participants in the IcoSema group and two participants in the semaglutide group discontinued treatment owing to gastrointestinal AEs. Gastrointestinal AEs were reported throughout the trial, with the proportion of affected individuals not exceeding 10% at any time during the trial for either treatment group (ESM Fig. 3).

Overall, the occurrence of AEs related to diabetic retinopathy (predefined Medical Dictionary for Regulatory Activities [MedDRA] search) from first dose to end of study was similar between treatment groups (IcoSema 7.6%; semaglutide 7.4%; event rate 0.08 events/person-years of observation with IcoSema vs 0.09 events/person-years of observation with semaglutide). All instances were non-serious and mild or moderate in severity. More events with the PT of diabetic retinopathy were reported in the IcoSema group than in the semaglutide group, in which the events were distributed across multiple preferred terms. No new safety concerns were identified for IcoSema in this trial, and the safety profile for IcoSema reflected that of the respective mono-components.

## Discussion

In individuals with type 2 diabetes in whom diabetes was inadequately managed by GLP-1 RA therapy with or without additional oral glucose-lowering medications, once-weekly IcoSema demonstrated statistical superiority to semaglutide 1.0 mg with respect to HbA_1c_ reduction from baseline to week 52. Additionally, IcoSema demonstrated similar rates of clinically significant hypoglycaemic episodes (there were no severe hypoglycaemic episodes in either group) and similar gastrointestinal tolerability when compared with semaglutide 1.0 mg; however, weight reduction was statistically significantly greater with semaglutide 1.0 mg than with IcoSema.

The 14.7 mmol/mol (1.35%-point) reduction in HbA_1c_ from baseline to week 52 seen in this trial with IcoSema was substantial. Interestingly, there was also a substantial reduction in HbA_1c_ in the semaglutide group (9.88 mmol/mol [0.90%-points]), which is surprising, given that the trial participants had all been receiving treatment with a GLP-1 RA; indeed, around half of the overall trial population had already been treated with semaglutide before enrolment in the trial. However, mean HbA_1c_ for this population was around 64.0 mmol/mol (8.0%), suggesting that either participants had not received appropriate treatment intensification/optimisation or that adherence to treatment prior to trial enrolment was poor. Notably, one-fifth of the participants randomised to semaglutide treatment were initiated on additional non-randomised insulin or non-insulin glucose-lowering medications lasting more than 2 weeks during the trial (vs only 3.8% in the IcoSema group). As such, the full impact of IcoSema on glycaemic management is greater than that illustrated by HbA_1c_ alone.

There was an initial, transient increase in mean pre-breakfast SMBG values during the first 3 weeks of treatment with IcoSema or semaglutide. This is most likely due to the discontinuation of the previous GLP-1 RA therapy and other oral glucose-lowering medications (DPP-4is, glinides and sulfonylureas) at randomisation, combined with the gradual up-titration of IcoSema or escalation of semaglutide from a lower initiating dose than participants were receiving before the trial. However, the increase in SMBG was minor (≤0.5 mmol/l), had reversed by approximately week 5–6 and, by the end of the trial, HbA_1c_, FPG and SMBG had improved in both treatment groups, suggesting that there was no lasting impact on glycaemic outcomes. Moreover, there was a significantly greater change in HbA_1c_ and FPG in favour of IcoSema when compared with semaglutide.

Over the treatment period, participants receiving semaglutide achieved a significant reduction in body weight, whereas participants receiving IcoSema experienced a mean weight gain of less than 1.0 kg by the end of the trial, probably caused by the basal insulin component of IcoSema. Weight gain associated with any basal insulin therapy is an important challenge. The weight gain associated with IcoSema treatment in this trial is modest, suggesting that the semaglutide component of IcoSema mitigated the weight gain normally expected with insulin initiation [18].

A key factor in managing type 2 diabetes with insulin is maintaining a balance between achieving good glycaemic control and minimising the risk of hypoglycaemia. Notably, the proportion of participants achieving HbA_1c_ <53 mmol/mol (<7.0%) without clinically significant or severe hypoglycaemia after 52 weeks was statistically significantly larger in the IcoSema group than in the semaglutide group, suggesting that IcoSema provides an appropriate balance between efficacy and hypoglycaemia risk.

Moreover, no new safety concerns were identified for IcoSema in this trial, with the observed safety profile for IcoSema reflecting those of icodec and semaglutide separately [3, 5–7, 9–12, 14, 15]. There were no severe hypoglycaemic episodes in either treatment group. No statistically significant difference was seen between IcoSema and semaglutide in the rate of clinically significant or severe hypoglycaemic episodes. These rates were low and comparable for both the IcoSema and semaglutide treatment groups, indicating that IcoSema has a similar hypoglycaemia rate to semaglutide, which is known to have a low hypoglycaemia risk profile [9–12, 14–16, 18].

Overall, the proportion of participants reporting gastrointestinal AEs was similar between the IcoSema and semaglutide treatment groups and was comparable with, or slightly lower than, those seen for semaglutide in the phase 3a SUSTAIN trials [9–11, 14]. As might be expected given that the trial participants had previously been treated with GLP-1 RAs, the proportion of participants experiencing gastrointestinal AEs remained low (<10%) throughout the trial.

There were comparable levels of AEs captured by the MedDRA PT of diabetic retinopathy in the IcoSema and semaglutide groups. Many of the occurrences of new onset or worsening diabetic retinopathy were detected only as part of the mandatory examination at week 52. It should be noted that the trial population as a whole had a long duration of diabetes and suboptimal glycaemic management at baseline, both of which are well-known risk factors for diabetic retinopathy. Moreover, individuals with controlled retinopathy (10.4% of the overall population) were permitted to participate in the trial. Participants who reported diabetic retinopathy across both groups generally had longer duration of diabetes (1–2 years) than those who did not report it, with most (86.3%) also having additional associated conditions, such as hypertension.

The findings reported here, with respect to IcoSema, are broadly in agreement with those seen with once-daily basal insulin and GLP-1 RA combination products. For example, superiority for change in HbA_1c_ from baseline to week 26 was demonstrated for once-daily IDegLira, a fixed-ratio combination therapy of insulin degludec with liraglutide, vs treatment with liraglutide alone [19]. Similarly, greater improvements were seen following treatment with iGlarLixi than with either insulin glargine U100 or lixisenatide alone [20]. By combining the attributes of GLP-1 RA with a basal insulin in a once-weekly formulation, IcoSema provides a good balance between efficacy and safety while also reducing the total number of injections in a year, compared with once-daily injectable therapies or separate administration of a once-weekly GLP-1 RA and a once-weekly basal insulin.

This was the first trial to assess outcomes for a once-weekly combination therapy of basal icodec and semaglutide in a large group of people with type 2 diabetes inadequately managed with GLP-1 RA therapy, with or without additional oral glucose-lowering medications. Moreover, the trial duration provides support for the long-term efficacy and safety of IcoSema in this population. However, there are some inherent limitations too. First, participants entered the trial at a low initiation dose for semaglutide (0.25 mg in the semaglutide group and 40 dose steps, equivalent to 0.114 mg semaglutide in the IcoSema group) and were then up-titrated over the course of several weeks. Consequently, the reduction in the dose of GLP-1 RA combined with the discontinuation of other oral glucose-lowering medications may have delayed the time taken to see an improvement in HbA_1c_. Second, the open-label trial design may have contributed to participant bias when reporting AEs. Third, RCT designs inherently have multiple clinician touchpoints for monitoring purposes, and this may not be reflective of routine practice. Finally, continuous glucose monitoring parameters were not captured in this trial, and might have provided useful additional insights.

### Conclusions

In people living with type 2 diabetes inadequately managed with GLP-1 RA therapy, with or without additional oral glucose-lowering medications, switching to once-weekly IcoSema, compared with once-weekly semaglutide 1.0 mg, demonstrated statistical superiority in HbA_1c_ reduction, similar rates of clinically significant or severe hypoglycaemic episodes and comparable gastrointestinal tolerability. However, weight change was statistically significant in favour of semaglutide 1.0 mg.

## Supplementary Information

Below is the link to the electronic supplementary material.

ESM (PDF 594 KB)

## Data Availability

Individual participant data will be shared in data sets in a de-identified/anonymised format. Shared data will include data sets from Novo Nordisk-sponsored clinical research completed after 2001 for product indications approved in both the EU and the USA. The trial protocol and redacted clinical trial report will be made available according to Novo Nordisk data sharing commitments. These data will be available after research completion and approval of product and product use in both the EU and the USA (no end date). Data will be shared with bona fide researchers submitting a research proposal requesting access to data, for use as approved by the Independent Review Board (IRB) according to the IRB charter (see novonordisk-trials.com). These data can be accessed via an access request proposal form; the access criteria can be found at novonordisk-trials.com. The data will be made available on a specialised SAS data platform. The results tables will be made available according to US and EU law, via Clinicaltrials.gov and EU Clinical Trials Register. Clinical trials synopsis will be uploaded to novonordisk-trials.com for clinical projects that have been discontinued.

## Abbreviations

AE: Adverse event
DPP-4i: Dipeptidyl peptidase-4 inhibitor
EOR: Estimated OR
ETD: Estimated treatment difference
FPG: Fasting plasma glucose
GLP-1 RA: Glucagon-like peptide-1 receptor agonist
IDegLira: Insulin degludec/liraglutide
IGlarLixi: Insulin glargine 100 U/ml plus lixisenatide
MedDRA: Medical Dictionary for Regulatory Activities
PT: Preferred term
PYE: Person-years of exposure
SMBG: Self-measured blood glucose
SOC: System organ class
