# Correction: Once-weekly IcoSema versus once-weekly semaglutide in adults with type 2 diabetes: the COMBINE 2 randomised clinical trial

**DOI:** 10.1007/s00125-025-06435-1

**Published:** 2025-05-05

**Authors:** Ildiko Lingvay, Malik Benamar, Liming Chen, Ariel Fu, Esteban Jódar, Tomoyuki Nishida, Jean-Pierre Riveline, Daisuke Yabe, Thomas Zueger, Rosângela Réa

**Affiliations:** 1 Endocrinology Division, Department of Internal Medicine and Peter O’Donnell Jr School of Public Health, University of Texas Southwestern Medical Center, Dallas, TX USA; 2 Novo Nordisk A/S, Søborg, Denmark; 3 NHC Key Laboratory of Hormones and Development, Tianjin Key Laboratory of Metabolic Diseases, Chu Hsien-I Memorial Hospital and Tianjin Institute of Endocrinology, Tianjin Medical University, Tianjin, China; 4 Universitary Hospital QuironSalud Madrid, Faculty of Biomedical and Health Sciences, Universidad Europea de Madrid, Madrid, Spain; 5 Novo Nordisk Pharma Ltd, Tokyo, Japan; 6 Department of Endocrinology and Diabetology, Lariboisiere Hospital, APHP, Paris-Cite University Paris, Paris, France; 7 Institut Necker Enfants Malades, Inserm U1151, CNRS UMR 8253, IMMEDIAB Laboratory, Paris, France; 8 Department of Diabetes, Endocrinology and Nutrition, Kyoto University Graduate School of Medicine, Kyoto, Japan; 9 Department of Endocrinology, Diabetes and Metabolic Diseases, Kantonsspital Olten, Olten, Switzerland; 10 Department of Diabetes, Endocrinology, Nutritional Medicine and Metabolism, Inselspital, Bern University Hospital, University of Bern, Bern, Switzerland; 11 Internal Medicine Department, Endocrine Division (SEMPR), Universidade Federal do Parana, Curitiba, Brazil

**Correction: Diabetologia (2025) 68:739-751**

10.1007/s00125-024-06348-5

The original analyses for fasting plasma glucose (FPG) and waist circumference were not aligned with what was pre-specified in the description of the estimand. The data have been reanalysed using the pre-specified on-treatment period instead of the in-study period, which has resulted in minor numerical changes:

The values for FPG in Table 2 and Fig. 2 have been corrected and the text has been updated where these data are mentioned.

The waist circumference values have been corrected in ESM Table 6.

In addition, the numerical values for the adverse events for IcoSema and semaglutide were incorrectly reported as number of events per 100 person-years of observation rather than per person-year of observation. These have been corrected.

These changes do not affect the statistical outcomes and conclusions, or the determination of clinically relevant impact.

The original article has been corrected.

**Table 2:** Summary of key efficacy and safety endpoints

Endpoint	LS mean		Treatment difference		
	IcoSema	Semaglutide 1.0 mg	Estimate	95% CI	*p* value
Estimated mean change in HbA_1c_ from baseline to week 52, mmol/mol	−14.7	−9.88	−4.85	−6.13, −3.57	<0.0001^a^
Estimated mean change in HbA_1c_ from baseline to week 52, %-points	−1.35	−0.90	−0.44	−0.56, −0.33	<0.0001^a^
Estimated mean change in FPG from baseline to week 52, mmol/l	−2.48	−1.41	−1.07	−1.37, −0.76	<0.0001
Estimated mean change in body weight from baseline to week 52, kg	0.84	−3.70	4.54	3.84, 5.23	<0.0001

^a^Primary endpoint. Superiority was confirmed based on the upper bound of the two-sided 95% CI treatment difference being strictly <0. Two-sided *p* values are presented. The change from baseline to week 52 in HbA_1c_, FPG and body weight was separately analysed using an ANCOVA model with region and randomised treatment as fixed factors and a baseline value as a covariate

LS, least-squares

**Fig. 2:**
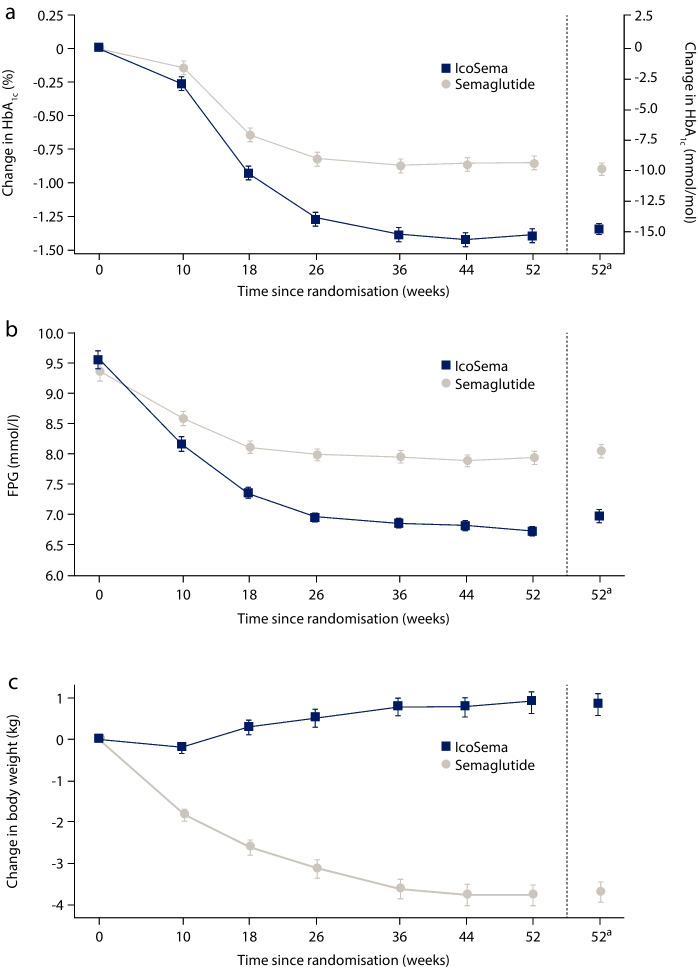
Observed key efficacy and safety outcomes in the full analysis set (IcoSema, *n*=342; semaglutide, *n*=341). (**a**) Change in HbA_1c_ from baseline, (**b**) change in FPG over time and (**c**) change in body weight from baseline. Data are presented as mean ± SEM. ^a^The estimated mean values and the corresponding SEM at week 52 were derived based on an ANCOVA model on multiple imputed data

