# Supplementary material for: Once-weekly IcoSema versus once-weekly semaglutide in adults with type 2 diabetes: the COMBINE 2 randomised clinical trial
Source: Diabetologia. 2025 Jan 17;68(4):739–51. doi: 10.1007/s00125-024-06348-5 (PMC11950020; doi:10.1007/s00125-024-06348-5)
Supplement: Supplementary file 1 — ESM (PDF 594 KB) [file 125_2024_6348_MOESM1_ESM.pdf]

Electronic supplementary materials

Contents

ESM Methods. Multiple imputation method applied for primary estimand analysis

ESM Table 1. Inclusion and exclusion criteria

ESM Table 2. Table of representativeness

ESM Table 3. Titration algorithm for weekly adjustment of IcoSema

ESM Table 4. Summary of the primary and secondary estimands

ESM Table 5. Changes to background glucose-lowering medications lasting more than 2 weeks from baseline until 1 week after the last dose of randomised treatment

ESM Table 6. Additional assessments: waist circumference and lipid parameters

ESM Fig 1. Overall trial design

ESM Fig 2. Pre-breakfast SMBG from over time from baseline to week 52

ESM Fig 3. Prevalence of gastrointestinal AEs

List of primary investigators and trial sites

ESM Methods

Multiple imputation method applied for primary estimand analysis

The multiple imputation approach used the following steps.

- Imputation: an ANCOVA model was applied for change in HbA_{1c} from baseline week 0 (V2) to week 52 for participants experiencing intercurrent events who had a measurement at week 52 with randomised treatment as fixed factor, last available planned on-treatment HbA_{1c} observation without initiation of non-randomised insulin treatment or additional non-insulin glucose-lowering medications for more than 2 weeks, the time point of last available planned on-treatment HbA_{1c} observation without initiation of non-randomised insulin treatment or additional treatments for more than 2 weeks and baseline HbA_{1c} as covariate. If participants not experiencing intercurrent events were missing measurements at week 52, a similar ANCOVA model was applied using available data from other participants who did not experience intercurrent events. The estimated parameters, and their variances, from the imputation models were used to impute missing HbA_{1c} measurements at week 52 1000 times to produce 1000 complete datasets.
- For each of the complete data sets, the primary endpoint was analysed using an ANCOVA model with region and randomised treatment as fixed factors, and baseline HbA_{1c} as covariate. The estimates and standard deviations for the 1000 data sets will be pooled to one estimate and associated standard deviation using Rubin's rule.
- From the pooled estimate and standard deviation, the 95% confidence interval for the treatment difference was calculated. The corresponding two-sided *p* value was also calculated.

This analysis was based on the underlying assumption that participants with missing data behave similarly to comparable participants within the same treatment arm, and similar for participants not experiencing intercurrent events.

ESM Table 1. Inclusion and exclusion criteria

**Inclusion criteria**
• Informed consent obtained before any trial-related activities. Trial-related activities are any procedures that are carried out as part of the trial, including activities to determine suitability for the trial.<br>• Male or female sex.<br>• Aged ≥18 years at the time of signing informed consent (≥20 years in Japan and Taiwan).<br>• Received a diagnosis of type 2 diabetes ≥180 days before the day of screening.<br>• Screening HbA_{1c} of 7.0–10.0% (53.0–85.8 mmol/mol), as measured by central laboratory.<br>• Insulin-naive. The following exceptions are permitted: short-term insulin treatment for a maximum of 14 days before screening and/or prior insulin treatment for gestational diabetes.<br>• Treated with stable doses of daily or weekly GLP-1 RA (excluding once-weekly semaglutide with doses higher than 1.0 mg) according to local label for the treatment of diabetes ≥90 days before screening. The treatment can be with or without any of the following oral glucose-lowering medications with stable doses ≥90 days before screening:<br>– metformin<br>– sulphonylurea<br>– metglinides (glinides)<br>– DPP-4is<br>– SGLT2is<br>– alpha-glucosidase inhibitors<br>– thiazolidinediones<br>– marketed oral combination products only including the products listed above.<br>• BMI ≤40.0 kg/m^2.<br>• Not currently using real-time continuous or flash glucose monitoring.
**Exclusion criteria**
• Known or suspected hypersensitivity to trial product(s) or related products.<br>• Previous participation in this trial. Participation is defined as giving signed informed consent.<br>• Individuals of female sex who are pregnant, breastfeeding or intending to become pregnant, or who are of childbearing potential and not using an adequate contraceptive method (adequate contraceptive measures as required by local regulation or practice).<br>• Participation in any clinical trial of an approved or non-approved investigational medicinal product in the 90 days before the day of screening.[a]

- Any disorder, except for conditions associated with type 2 diabetes, that in the investigator's opinion might jeopardise the participant's safety or compliance with the protocol.
- Anticipated initiation or change in concomitant medications (for >14 consecutive days) known to affect weight or glucose metabolism (e.g. treatment with orlistat, thyroid hormones or corticosteroids).
- Treatment with any medication for the indication of diabetes or obesity other than those stated in the inclusion criteria in the 90 days before the day of screening.
- Any episodes[b] of diabetic ketoacidosis within 90 days before the day of screening.
- Personal or first-degree relative(s) history of multiple endocrine neoplasia type 2 or medullary thyroid carcinoma.
- Presence or history of pancreatitis (acute or chronic) in the 180 days before the day of screening.
- Any of the following: myocardial infarction, stroke, hospitalisation for unstable angina pectoris, or transient ischaemic attack in the 180 days before the day of screening.
- Chronic heart failure classified as being in New York Heart Association class IV at screening.
- Planned coronary, carotid or peripheral artery revascularisation.
- Renal impairment with estimated glomerular filtration rate value of <30 mL/min/1.73 m^2 at screening, as defined by KDIGO 2012.
- Impaired liver function, defined as alanine aminotransferase ≥2.5 times or bilirubin >1.5 times the upper normal limit at screening, as measured by central laboratory.
- Inadequately treated blood pressure defined as systolic pressure of ≥180 mm Hg or diastolic pressure of ≥110 mm Hg at screening.
- Uncontrolled and potentially unstable diabetic retinopathy or maculopathy, verified by a fundus examination performed in the 90 days before the day of screening or in the period between screening and randomisation. Pharmacological pupil dilation is a requirement unless using a digital fundus photography camera specified for non-dilated examination.
- Presence or history of malignant neoplasm (other than basal or squamous cell skin cancer, in situ carcinomas of the cervix or in situ prostate cancer) in the 5 years before the day of screening.

[a]Simultaneous participation in a trial with the primary objective of evaluating an approved or non-approved investigational medicinal product for prevention or treatment of COVID-19 disease or post-infectious conditions is allowed if the last dose of the investigational medicinal product has been received more than 30 days before screening.

[b]As declared by the participant or in the medical records.

DPP-4i, dipeptidyl peptidase-4 inhibitor; GLP-1 RA, glucagon-like peptide-1 receptor agonist; KDIGO 2012, Kidney Disease: Improving Global Outcomes 2012 clinical practice guidelines; SGLT2i, sodium–glucose cotransporter 2 inhibitor.

ESM Table 2. Representativeness of study participants

Category: Type 2 diabetes

Special considerations related to	
Sex and gender	The global prevalence of T2D is slightly higher in men than women.[1,2]
	The diagnosis of T2D is usually made at a younger age in men than women.[2]
Age	Although T2D can occur at any age, prevalence increases with age. Based on prevalence data from the National Health Interview Survey (NHIS) in 2016-2017, prevalence reaches of 11.1% adults 45–64 years of age, with the highest prevalence observed in those >65 years of age (18.2% of all adults).[3]
Race or ethnic group	Prevalence of T2D is higher in Asian, Black and Hispanic groups compared with White populations.[4,5]
Geography	There is substantial variation in prevalence and incidence of T2D globally. Some of the highest prevalence rates are in USA, Mexico, island nations in the Pacific Ocean, South-east Asia and Western Europe.[6]
Socioeconomic background	This was not collected as part of this trial.
Other considerations	Individuals with higher BMI are more likely to have or develop T2D,8 though men are usually diagnosed with lower body fat mass than women.[2]
Overall representativeness of this trial	The participant sample in this trial is largely representative of clinical settings and of the intended study population (i.e. those already treated with GLP-1 RA) with slightly more men than women included (57.5–58.8% across treatment arms) and a mean age of ~59 years. The observed mean BMI in this study was approximately 31 kg/m^2 across treatment arms. Moreover, the study population covered multiple races, and both Hispanic or Latino ethnicities were also included. This was a multiregional study and included those countries/regions with

	some of the highest prevalence (Western Europe, USA, mainland China and Brazil).

Note: Sex, race, and ethnicity were collected using multiple choice questions on an electronic case report form completed by the study sites at enrollment. Sex, race and ethnicity were reported by the participant or their legally authorized representative. Sex was reported as Male o Female. Race was reported as White, Black/African American, Asian, Native Hawaiian/Other Pacific Islander, American Indian/Alaskan Native, or more than one race. Ethnicity was reported as Hispanic/Latino or not Hispanic/Latino.

1. Huebschmann, A.G.*, et al.* Sex differences in the burden of type 2 diabetes and cardiovascular risk across the life course. *Diabetologia* **62**, 1761-1772 (2019).

2. Kautzky-Willer, A., Leutner, M. & Harreiter, J. Sex differences in type 2 diabetes. *Diabetologia* **66**, 986-1002 (2023).

3. Xu, G.*, et al.* Prevalence of diagnosed type 1 and type 2 diabetes among US adults in 2016 and 2017: population based study. *BMJ* **362**, k1497 (2018).

4. Rodriguez, J.E. & Campbell, K.M. Racial and Ethnic Disparities in Prevalence and Care of Patients With Type 2 Diabetes. *Clin Diabetes* **35**, 66-70 (2017).

5. Pham, T.M., Carpenter, J.R., Morris, T.P., Sharma, M. & Petersen, I. Ethnic Differences in the Prevalence of Type 2 Diabetes Diagnoses in the UK: Cross-Sectional Analysis of the Health Improvement Network Primary Care Database. *Clin Epidemiol* **11**, 1081-1088 (2019).

6. IDF Diabetes Atlas 2021 10th edition. Available at: https://diabetesatlas.org/idfawp/resource-files/2021/07/IDF_Atlas_10th_Edition_2021.pdf

ESM Table 3. Titration algorithm for weekly adjustment of IcoSema

	**Pre-breakfast SMBG**		**Once-weekly IcoSema dose adjustment**
Value to use	mmol/L	mg/dL	Dose steps
**Mean** of the SMBG values	>7.2	>130	+10
	4.4−7.2	80−130	0
**Lowest** of the SMBG values	<4.4	<80	−10

Weekly dose adjustment was based on three pre-breakfast SMBG values measured 2 days before titration and on the day of titration. If one or more SMBG values were missing, the dose adjustment was performed based on the remaining values.

ESM Table 4. Description of the estimands

Estimand	Description
Primary estimand	*Change in HbA_{1c} from baseline to week 52*<br>• Treatment condition: the effect of once-weekly IcoSema vs fixed dose escalation of once-weekly semaglutide 1.0 mg (with or without oral glucose-lowering medications), regardless of adherence to randomised treatment or initiation of non-randomised insulin or additional non-insulin glucose-lowering medications for more than 2 weeks.<br>• Population: type 2 diabetes inadequately controlled with a GLP-1 RA.<br>• Endpoint: change in HbA_{1c} from baseline to week 52.<br>• Remaining ICEs: none. The two ICEs are captured under treatment condition and handled as follows.<br>– Initiation of non-randomised insulin treatment or additional non-insulin glucose-lowering medications for more than 2 weeks by the treatment policy strategy.<br>– Discontinuation of randomised treatment for any reason by the treatment policy strategy.<br>• Population-level summary: difference in mean changes from baseline.
Secondary estimands	*Change in body weight from baseline to week 52*<br>• Treatment condition: the effect of once-weekly IcoSema vs fixed dose escalation of once-weekly semaglutide 1.0 mg (with or without oral glucose-lowering agents), regardless of adherence to randomised treatment or initiation of non-randomised insulin or additional non-insulin glucose-lowering medications for more than 2 weeks.<br>• Population: type 2 diabetes inadequately controlled with a GLP-1 RA.<br>• Endpoint: change in body weight from baseline to week 52.<br>• Remaining ICEs: none. The two ICEs are captured under treatment condition and handled as follows.<br>– Initiation of non-randomised insulin treatment or additional non-insulin glucose-lowering medications for more than 2 weeks by the treatment policy strategy.<br>– Discontinuation of randomised treatment for any reason by the treatment policy strategy.<br>• Population-level summary: difference in mean changes from baseline.<br>*FPG levels from baseline to week 52*

Estimand	Description
	• Treatment condition: the effect of once-weekly IcoSema vs fixed dose escalation of once-weekly semaglutide 1.0 mg (with or without oral glucose-lowering medications), regardless of adherence to randomised treatment or initiation of non-randomised insulin or additional non-insulin glucose-lowering medications for more than 2 weeks, assuming all participants adhered to randomised treatment until week 52.<br>• Endpoint: change in FPG from baseline to week 52.<br>• Population: type 2 diabetes inadequately controlled with a GLP-1 RA.<br>• Remaining ICEs: none. The two ICEs are captured under treatment condition and handled as follows.<br>– Initiation of non-randomised insulin treatment or other non-insulin glucose-lowering medications for more than 2 weeks by the treatment policy strategy.<br>– Discontinuation of randomised treatment for any reason by the hypothetical strategy.<br>• Population-level summary: difference in mean.<br>*Numbers of clinically significant (level 2) or severe (level 3) hypoglycaemic episodes from baseline to week 57*<br>• Treatment condition: the effect of once-weekly IcoSema vs fixed dose escalation of once-weekly semaglutide 1.0 mg (with or without oral glucose-lowering medications), regardless of adherence to randomised treatment or initiation of non-randomised insulin or additional non-insulin glucose-lowering medications for more than 2 weeks, assuming all participants adhered to randomised treatment until week 52.<br>• Population: type 2 diabetes inadequately controlled with a GLP-1 RA.<br>• Endpoint: number of clinically significant (level 2) or severe (level 3) hypoglycaemic episodes from baseline to week 57.<br>• Remaining ICEs: none. The two ICEs are captured under treatment condition and handled as follows.<br>– Initiation of non-randomised insulin treatment or other non-insulin glucose-lowering medications for more than 2 weeks by the treatment policy strategy.<br>– Discontinuation of randomised treatment for any reason by the hypothetical strategy.<br>• Population-level summary: rate ratio.

Estimand	Description
	Numbers of clinically significant hypoglycaemic episodes from baseline to week 57<br>• Treatment condition: the effect of once-weekly IcoSema vs fixed dose escalation of once-weekly semaglutide 1.0 mg (with or without oral glucose-lowering medications), regardless of adherence to randomised treatment or initiation of non-randomised insulin or additional non-insulin glucose-lowering medications for more than 2 weeks, assuming all participants adhered to randomised treatment until week 52.<br>• Population: type 2 diabetes inadequately controlled with a GLP-1 RA.<br>• Endpoint: number of clinically significant (level 2) hypoglycaemic episodes from baseline to week 57.<br>• Remaining ICEs: none. The two ICEs are captured under treatment condition and handled as follows.<br>– Initiation of non-randomised insulin treatment or other non-insulin glucose-lowering medications for more than 2 weeks by the treatment policy strategy.<br>– Discontinuation of randomised treatment for any reason by the hypothetical strategy.<br>• Population-level summary: rate ratio.<br>*Numbers of severe hypoglycaemic episodes from baseline to week 57*<br>• Treatment condition: the effect of once-weekly IcoSema vs fixed dose escalation of once-weekly semaglutide 1.0 mg (with or without oral glucose-lowering medications), regardless of adherence to randomised treatment or initiation of non-randomised insulin or additional non-insulin glucose-lowering medications for more than 2 weeks, assuming all participants adhered to randomised treatment until week 52.<br>• Population: type 2 diabetes inadequately controlled with a GLP-1 RA.<br>• Endpoint: number of severe hypoglycaemic episodes from baseline to week 57.<br>• Remaining ICEs: none. The two ICEs are captured under treatment condition and handled as follows.<br>– Initiation of non-randomised insulin treatment or other non-insulin glucose-lowering medications for more than 2 weeks by the treatment policy strategy.<br>– Discontinuation of randomised treatment for any reason by the hypothetical strategy.<br>• Population-level summary: rate ratio.

Estimand	Description

FPG, fasting plasma glucose; GLP-1 RA, glucagon-like peptide-1 receptor agonist; ICE, intercurrent event; IcoSema, a once-weekly combination therapy of basal insulin icodec and semaglutide.

ESM Table 5. Changes to background glucose-lowering medications lasting more than 2 weeks from baseline until 1 week after the last dose of randomised treatment

	**IcoSema (*n*=342)**	**Semaglutide (*n*=341)**
**Initiation of non-randomised insulin treatment or additional non-insulin glucose-lowering medications**		
Overall	13 (3.8)	71 (20.8)
Non-insulin glucose-lowering medication, n (%)	12 (3.5)	64 (18.8)
SGLT2i	5 (1.5)	18 (5.3)
Sulphonylurea	4 (1.2)	19 (5.6)
Alpha-glucosidase inhibitor	1 (0.3)	3 (0.9)
DPP-4i	1 (0.3)	1 (0.3)
Metformin	1 (0.3)	14 (4.1)
Thiazolidinediones	1 (0.3)	16 (4.7)
Glinides	0	1 (0.3)
GLP-1 RA	0	1 (0.3)
Basal insulin	1 (0.3)	12 (3.5)
Insulin degludec	1 (0.3)	6 (1.8)
Insulin glargine U100	0	5 (1.5)
Insulin glargine U300	0	1 (0.3)
Bolus insulin	0	3 (0.9)
Insulin aspart	0	2 (0.6)
Insulin lispro	0	1 (0.3)

Only changes lasting more than 2 weeks are included.

%, proportion of participants; DPP-4i, dipeptidyl peptidase-4 inhibitor; GLP-1 RA, glucagon-like peptide-1 receptor agonist; SGLT2i, sodium–glucose cotransporter 2 inhibitor; SU, sulphonylureas.

ESM Table 6. Additional assessments: waist circumference, blood pressure and lipid parameters

**Assessment**	**Estimate**		**IcoSema vs semaglutide 1.0 mg, (95% CI)**
	**IcoSema (*n*=342)**	**Semaglutide 1.0 mg (*n*=341)**	
Change in waist circumference from baseline to week 52, cm	1.17	–3.47	ETD: 4.63 (3.78, 5.49); *p*<0.0001
Change in systolic blood pressure from baseline to week 52, mm Hg	–0.42	–3.06	ETD: 2.64 (0.72, 4.55) *p*=0.0069
Change in diastolic blood pressure from baseline to week 52, mm Hg	0.23	–1.06	ETD: 1.29 (0.16, 2.42) *p*=0.0254
Relative change in HDL cholesterol in serum from baseline to week 52	1.06	1.08	ETR: 0.98 (0.96, 1.01) *p*=0.1406
Relative change in LDL cholesterol in serum from baseline to week 52	0.99	0.98	ETR: 1.01 (0.95, 1.06) *p*=0.8393
Relative change in VLDL cholesterol in serum from baseline to week 52	0.81	0.87	ETR: 0.93 (0.88, 0.99) *p*=0.0231
Relative change in triglycerides in serum from baseline to week 52	0.82	0.88	ETR: 0.93 (0.88, 0.99) *p*=0.0217
Relative change in total cholesterol in serum from baseline to week 52	0.97	0.98	ETR: 0.99 (0.96, 1.02) *p*=0.4029
Relative change in free fatty acids in serum from baseline to week 52	0.74	0.88	ETR: 0.84 (0.78, 0.90) *p*<0.0001

Data are estimated LS means. *P* value: two-sided *p* value for test of no treatment difference. No correction for multiplicity. The changes in systolic blood pressure, diastolic blood pressure and waist circumference from baseline to week 52 were analysed using an ANCOVA model with region and randomised treatment as fixed factors and a baseline value as a covariate. For lipid parameters, the log-transformed response after 52 weeks was analysed using an ANCOVA model with region and randomised treatment as fixed factors and the log-transformed baseline response as a covariate.
ETD, estimated treatment difference; LS, least-squares.

ESM Fig. 1 Overall trial design

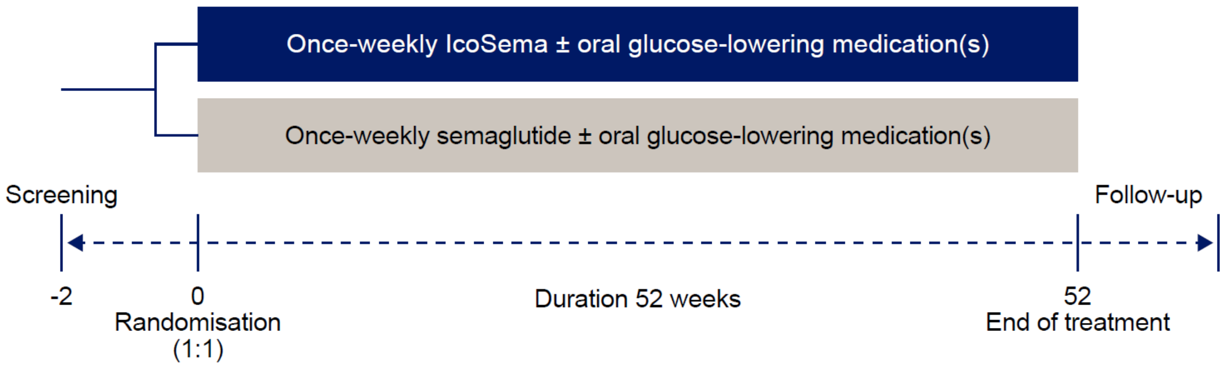

ESM Fig. 2 Pre-breakfast SMBG over time from baseline to week 52

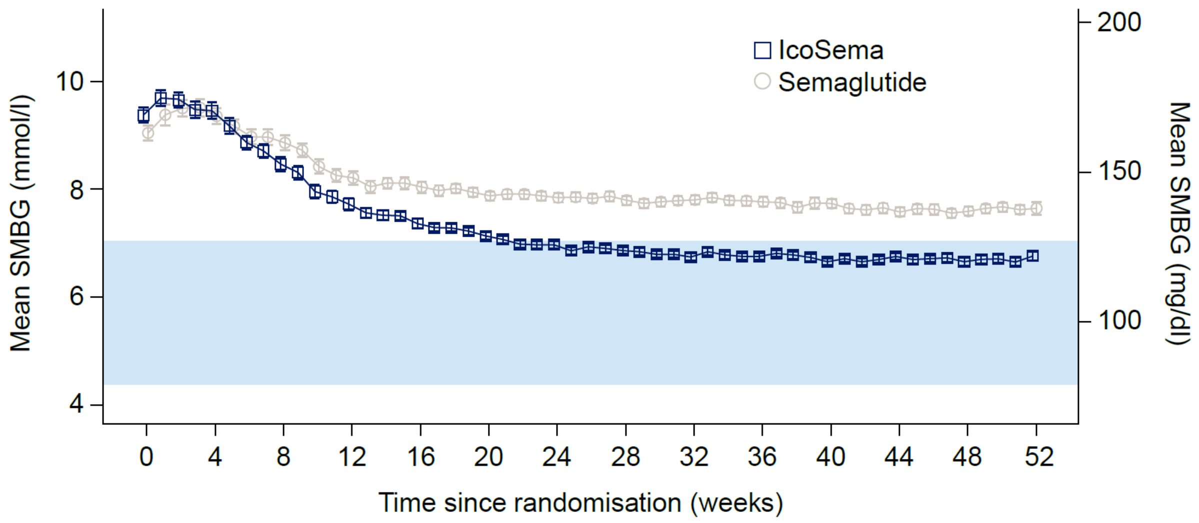

Data are mean SMBG of the last 3 days of each week (symbol) ± SEM (error bars). Blue panel represents the target range (4.4–7.2 mmol/L [80–130 mg/dL]).

Observed data including data obtained after premature treatment discontinuation.

SMBG, self-measured blood glucose.

ESM Fig. 3 Prevalence of gastrointestinal AEs

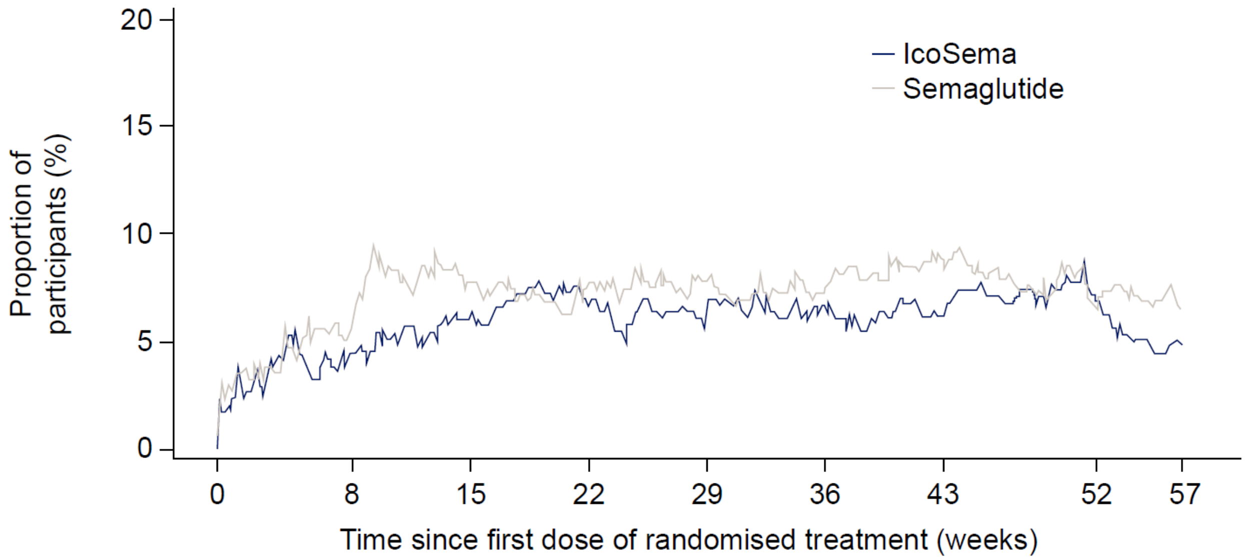

Safety analysis set. Events during the on-treatment period, defined as an onset date on or after the first dose of randomised treatment and no later than the first date of: the last follow-up visit, the last date on randomised treatment +6 weeks (corresponding to 5 weeks after the end of the dosing interval for both treatment groups) or the end date for the in-trial points sets.

AE, adverse event.

List of primary investigators and trial sites

Country/ Region	Site name	Site address	Name of primary investigator	No. screened	No. randomised
USA	South Broward Research LLC	3000 SW 148 Ave<br>Suite 112<br>33027 Miramar<br>Florida	Kenneth Blaze	1	1
USA	Clinical Research Institute of Arizona	14506 W Granite Valley Dr<br>Suite 214<br>85375 Sun City West<br>Arizona	Jalal Abbas	4	3
USA	Osvaldo A. Brusco MD PA	5814 Esplanade Drive<br>78414 Corpus Christi<br>Texas	Osvaldo Brusco	8	7
USA	Florida Inst For Clin Res	10967 Lake Underhill Road Suite 117<br>32825 Orlando<br>Florida	Humberto Cruz	7	4
USA	New Venture Medical Research	251 Leatherman Road<br>44281 Wadsworth<br>Ohio	Matthew Finneran	1	1
USA	Northern Pines Hlth Ctr	PC 11293 N. M37<br>Suite A<br>49620 Buckley<br>Michigan	Mitzie Hewitt	2	2
USA	Adult Medicine of Lake County	Inc. 3587 Lake Center Drive<br>32757 Mount Dora<br>Florida	Shirley Nagel	9	6
USA	Palm Research Center Inc-Vegas	9280 West Sunset Road<br>Suite 306<br>89148 Las Vegas<br>Nevada	Samer Nakhle	6	4
USA	Albuquerque Clin Trials, Inc.	711 Encino Place NE<br>87102 Albuquerque<br>New Mexico	Marigene Sharma	4	1
USA	Northeast Research Institute	1635 Eagle Harbor Parkway<br>Suite 6<br>32003 Fleming Island<br>Florida	Khurram Wadud	13	10

USA	Chrysalis Clinical Research	736 S. 900 E.<br>Suite 201<br>84790 St. George<br>Utah	Joseph Woolley	6	5
USA	NE Clin Res of San Antonio	7323 N Loop 1604 E<br>Suite 503<br>78233 San Antonio<br>Texas	Laura Akright	5	5
USA	Amarillo Med Spec LLP	1215 South Coulter St.<br>Suite 402<br>79106 Amarillo<br>Texas	William Biggs	6	6
USA	Palmetto Clinical Research	1516 Old Trolley Rd<br>Suite 100<br>29485 Summerville<br>South Carolina	D. Eric Bolster	5	3
USA	Texas Diabetes & Endocrinology	5000 Davis Lane<br>Suite 200<br>78749 Austin<br>Texas	Tira Chaicha-Brom	3	3
USA	John Muir Physician Network	2700 Grant St<br>Suite 202<br>94520 Concord<br>California	Anna Chang	1	1
USA	Valley Clinical Trials, Inc.	18433 Roscoe Blvd.<br>Suite 203, 208, & 210<br>91325 Northridge<br>California	Christopher Chow	4	4
USA	Northeast Res Inst. Inc.	915 W Monroe St, Suite 200<br>32204 Jacksonville<br>Florida	John Evans	5	4
USA	Diab & Endo Assoc of Stark Co	4565 Dressler Rd NW<br>Ste 111<br>44718 Canton<br>Ohio	Arvind Krishna	4	3
USA	PharmQuest Life Sciences LLC	806 Green Valley Road<br>Suite 305<br>27408 Greensboro<br>North Carolina	Alexander Murray	9	7
USA	MedStar Hlth Res Institute	6525 Belcrest Road<br>Suite 700<br>20782 Hyattsville<br>Maryland	Jean Park	2	1
USA	East West Med Res Inst	1585 Kapiolani Blvd.<br>Suite 1500<br>96814 Honolulu<br>Hawaii	Cindy Pau	8	6

USA	Texas Diabetes & Endocrinology_Round Rock	110 Deer Ridge Drive<br>78681 Round Rock<br>Texas	Hien Tran	3	3
USA	DCOL Ctr for Clin Res	707 Hollybrook Drive<br>Suite 501<br>75605 Longview<br>Texas	Kathleen Harris	5	3
USA	Javara Inc/Privia Md Gp LLC Fst	1111 Corporate Park Drive<br>Suite C<br>24551 Forest<br>Virginia	Mark Kleiner	4	3
USA	AM Diabetes And Endocrinology Center	3025 Kate Bond Road<br>38133 Bartlett<br>Tennessee	Kashif Latif	1	1
USA	Est Cst Inst for Rsrch,Jksnvil	3550 University Blvd S.<br>Suite 101<br>32216 Jacksonville<br>Florida	Mae Sheikh-Ali	6	3
USA	Saltzer Medical Group Research	215 E Hawaii Ave<br>Suite 240<br>83686-6011 Nampa<br>Idaho	Stanley Stringam	3	1
USA	NorthShore Univ Hlth Sys	9977 Woods Drive<br>Ste 300<br>60077 Skokie<br>Illinois	Liana Billings	5	5
USA	North Texas Endocrine Center	9301 North Central Expressway<br>Suite 570<br>75231 Dallas<br>Texas	Peter Bressler	4	4
USA	Chattanooga Medical Research, LLC	2341 McCallie Ave<br>Suite 300<br>37404 Chattanooga<br>Tennessee	Charles Crump	4	3
USA	Velocity Clin Res Wstlke	2010 Wilshire Blvd.<br>Ste #302<br>90057 Los Angeles<br>California	Juan Pablo Frias	3	3
USA	Thomas Jefferson Univ	Diabetes Research Center<br>211 South 9th Street<br>Suite 600<br>19107 Philadelphia<br>Pennsylvania	Kevin J. Furlong	2	2

USA	Univ of AL at Birmingham_BRM	1675 University Blvd., Room 256<br>35294 Birmingham<br>Alabama	William Garvey	5	3
USA	Javara/Privia Med Grp GA,LLC	2402 Osler Court<br>31707 Albany<br>Georgia	Charles Kemp	5	4
USA	Premier Medical Center, Inc.	4418 Vineland Avenue<br>Suite 102<br>91602 Toluca Lake<br>California	Michael Marsh	4	0
USA	Downtown LA Res Ctr. Inc.	1055 Wilshire Blvd<br>Suite 1660<br>90017 Los Angeles<br>California	Salil Nadkarni	1	0
USA	TPMG Clinical Research	860 Omni Boulevard<br>Suite 101<br>23606 Newport News<br>Virginia	Linda Schneider	8	7
USA	John J Shelmet, MD	3131 Princeton Pike, Building 2B Suite #104<br>08648 Lawrenceville<br>New Jersey	John Shelmet	4	4
USA	Southgate Medical Group, LLP	1026 Union Road<br>14224 West Seneca<br>New York	Brian Snyder	3	3
USA	Diabetes and Thyroid Ctr of FW<br>6844 Harris Pkway<br>Suite 300<br>76132<br>Fort Worth<br>Texas	1026 Union Road<br>14224 West Seneca<br>New York	Anjanette Tan	3	3
USA	Ileana J Tandron APMC	2240 Gause Blvd E<br>70461-4231 Slidell<br>Louisiana	Ileana Tandron	4	2
USA	Metabolic Research Institute Inc	1515 N. Flagler Drive<br>Suite 440<br>33401 West Palm Beach<br>Florida	William Kaye	10	6
USA	Oviedo Medical Research, LLC	2441 W. State Road 426<br>Suite 2011<br>32765 Oviedo<br>Florida	Bradley Block	3	2

USA	Texas Diab & Endo, P.A.	6500 N. Mopac<br>Building 3, Suite 200<br>78731 Austin<br>Texas	Valerie Espinosa	4	3
USA	Sugar Lakes Family Practice PA	16902 Southwest Fwy<br>Suite 100<br>77479 sugar land<br>Texas	John Vanderzyl	9	4
USA	Pri Med Grp dba/Gil Ctr Fam Gilbert Ctr for Family Medicine	652 E. Warner Rd<br>Ste 107<br>85296 Gilbert<br>Arizona	Sunildat Maheshwari	6	4
USA	UT Southwestern Med Cntr	5323 Harry Hines Blvd U9.134B<br>75390-9302 Dallas<br>Texas	Ildiko Lingvay	9	8
Taiwan	National Cheng Kung University Hospital	10A in-patient building<br>No.138,Sheng Li Road<br>704 Tainan City	Horng-Yih Ou	5	4
Taiwan	Chi Mei Medical Center	No.901, Zhonghua Rd. YongKang Dist.<br>710 Tainan City	Kai-Jen Tien	5	4
Taiwan	Chung Shan Medical University Hospital	No. 110, Sec. 1, Jianguo N. Rd<br>402 Taichung City	Chien-Ning Huang	5	4
Taiwan	Chang Gung Medical Foundation - Linkou Branch	No.5 Fu-Shin Street, Kweishan Dist.<br>333 Taoyuan city	Yu-Yao Huang	4	3
Taiwan	Taipei Veterans General Hospital	No. 201, Shih-Pai Rd, Sec 2<br>112 Taipei	Chii-Min Hwu	5	5
Slovakia	Diabetologicka ambulancia DIADAN, s.r.o.	Kosice Brigadnicka 2<br>04011 Kosice	Dana Solcova	7	7
Slovakia	DIOLI s.r.o.	Ambulancia diabetologie a poruch latkovej premeny a vyzivy<br>Narodna Trieda 27<br>040 01 Kosice	Olga Bobelova	9	7
Slovakia	FNsP L. Pasteura	Rastislavova 43<br>04190 Kosice	Martin Javorsky	4	4
Slovakia	DIA - KONTROL s.r.o.	Ambulancia diabetologie, poruchy latkovej premeny a vyzivy<br>SNP 19<br>93401 Levice	Anna Vargova	12	8

Slovakia	MUDr. Alena Lomencikova, s.r.o	9. maja 438/9<br>039 01 Turcianske Teplice	Alena Lomencikova	10	9
Slovakia	HUMAN-CARE s.r.o.	diabetologicka ambulancia<br>Rastislavova 45<br>040 01 Kosice	Viera Donicova	13	11
Sweden	Centrum for Diabetes, Academical Specialist Centrum	Solnavägen 1E<br>113 65 Stockholm	Sergiu-Bogdan Catrina	1	1
Sweden	Universitetssjukhuset i Örebro	Enheten för Kliniska Studier (EKS)<br>S-huset, entré S2, BV<br>Södra Grev Rosengatan 52C<br>703 62 Örebro	Ken Eliasson	9	5
Sweden	Sahlgrenska Sjukhuset	Primary Care Trial Center, PTC<br>Gothia Forum<br>Gröna stråket 12, plan 0<br>413 46 Göteborg	Dan Curiac	7	3
Japan	Shimizu Clinic Fusa	5-46-7, Misono, Midori-ku, Saitama-shi<br>336-0967 Saitama	Yukari Shimizu	12	10
Japan	The Institute of Medical Science, Asahi Life Foundation	2-2-6 Nihonbashi Bakurocho,<br>103-0002 Chuo ku Tokyo	Yukiko Onishi	16	15
Japan	Takatsuki Red Cross Hospital	1-1-1, Abuno, Takatsuki-shi<br>569-1045 Osaka	Haruhiko Onaka	13	13
Japan	Heiwadai Hospital	150-1, Yanosakicho<br>880-0034 Miyazaki-shi<br>Miyazaki	Shuji Nakamura	13	12
Japan	Naka Kinen Clinic	745-5, Nakadai, Naka-shi<br>311-0113 Ibaraki	Takeshi Osonoi	19	19
Japan	Futata Tetsuhiro Clinic Meinohama	1-2-17, Meinohamaeki-minami, Nishi-ku<br>819-0006 Fukuoka-shi<br>Fukuoka	Dai Shimono	18	17
Japan	Gifu University Hospital 1-1	Yanagito, Gifu-shi,<br>501-1194 Gifu	Takehiro Kato	2	2
Japan	Shinden Higashi Clinic	2-10-4 Shindenhigashi, Miyagino-ku<br>983-0039 Sendai-shi<br>Miyagi	Fumiki Oh	11	8

Japan	Soka Sugiura Internal Medicine Clinic_Internal Medicine	Soka SKbiru2F 2-11-23 Takasago, 340-0015 Soka-shi	Tatsushi Sugiura	4	4
Israel	Diabetes Unit Hadassah Ein Karem MC	Kiryat Hadassa street 91120 Jerusalem	Ofri Mosenzon	10	9
Israel	Endrocrinolgy Clinic - Sheba Medical Center Endrocrinolgy, metabolism & diabetic center	Clinical research unit. 1 Emek Ha'ela street 52621 Tel Hashomer	Amir Tirosh	7	6
Israel	Diabetes Clinic Wolfson MC	Outpatient diabetic clinic 58100 Holon	Julio Wainstein	8	7
Israel	Linn clinic - Clalit Health Services Linn Medical Center Clalit Sick Fund	Research Unit, Diabetes and Lipids Department 35 Rothschild Boulevard 35152 Haifa	Muhammad Sabbah	7	6
Israel	Clalit sick fund	Herzlia Harava 23 4630945 Herzlia	Eytan Roitman	9	6
Israel	Diabetes Clinic Meir Meir Medical center	Clinical Research Unit Stolberg building, 5th floor 44281 Kfar Saba	Victor Vishlitzky	1	1
Greece	Iatriko Psychicou Private Clinic	Department of Internal Medicine & Diabetes 1, Andersen Str. Psychico 115 25	Gerasimos Karousos	15	11
Greece	General Hospital of Thessaloniki "G.Papanikolaou"	A' Internal Medicine Clinic Diabetes Center	Iakovos Avramidis	14	13
Greece	Univ Gen Hospital Larisa, Mezourlo area	Clinic of Endocrinology and Metabolic Disease Building C’ 2nd floor	Alexandra Bargiota	9	8
Greece	"Ippokrateio" G.H. of Thessaloniki	Diabetology Clinic 49, Konstantinoupoleos str.	Ioanna Zografou	16	14
Greece	General Hospital of Thessaloniki 'G. Gennimatas	41 Ethnikis Aminis str. 54635 Thessaloniki	Ioannis Stergiou	5	5
Greece	G. Gennimatas' General Hospital of Athens	154 Mesogion Ave. Cholargos 115 27	Athina Markou	8	8
Greece	Evangelismos Hospital	45-47 Ipsilantou Str. 10676 Athens	Dimitra Argyro Vassiliadi	11	8

Greece	"Thermi" Private Hospital	Department of Internal Medicine 14km N.R. Thessaloniki - N. Moudania,Thermi	Emmanouil Pagkalos	6	5
France	GROUPE HOSPITALIER MUTUALISTE DES PORTES DU SUD	Service d'Endocrinologie-Diabétologie-Maladies Métaboliques 2 av du 11 novembre 1918 69200 Venissieux	Lucien Marchand	24	22
France	CENTRE HOSPITALIER UNIVERSITAIRE DE NANTES-HOPITAL NORD LAENNEC	1 Boulevard Jacques MONOD 44800 SAINT HERBLAIN	Bertrand Cariou	3	3
France	GROUPE SOS SANTE–HOPITAL LE CREUSOT-HOTEL DIEU Site Harfleur	1 Service de Diabétologie 26 rue d'Harfleur 71200 Le Creusot	Sylvaine CLAVEL	5	4
France	CENTRE HOSPITALIER UNIVERSITAIRE DE TOULOUSE-HOPITAL RANGUEIL	2 Diabétologie - Maladies Métaboliques - Nutrition 1, Avenue Jean Poulhes 31054 TOULOUSE	Pierre Gourdy	4	4
France	LES HOPITAUX DE CHARTRES-HOPITAL LOUIS PASTEUR	Service de diabétologie-endocrinologie-nutrition Cardiologie 5 Etage 2 4, rue Claude Bernard 28630 Le Coudray	Arnaud Monier	2	2
Mainland China	Peking University People's Hospital	No.11 Xizhimen South Street, Xicheng District 100044 Beijing Beijing	Linong JI	2	1
Mainland China	Shanghai Fifth People's Hospital	No.128, Ruili Road, Minhang District 200240 Shanghai Shanghai	Jun LIU	4	4
Mainland China	General Hospital of Tianjin Medical University	No. 154 Anshan Road, Heping District 300052 Tianjin Tianjin	Ming LIU	7	6

Mainland China	Jinan Central Hospital	No.105, Jiefang Road, Lixia District 250013 Jin‘an Shandong	Xiaolin DONG	14	11
Mainland China	The Second Affiliated Hospital of Nanjing Medical University_Nanjing	No. 121 Jiangjiayuan Gulou District 210011 Nanjing Jiangsu	Yibing LU	13	11
Mainland China	Changzhou No.2 People’s Hospital, Yanghu Branch	No.68, Middle Gehu Road, Wujin District 213003 Changzhou Jiangsu	Xinhua YE	2	1
Mainland China	Chongqing University Three Gorges Hospital	No.165 XinCheng Road Wanzhou District 404000 ChongQing Chongqing	Yong LUO	3	3
Mainland China	Huizhou Central People's Hospital	No.41, E ling north Road 516001 Huizhou Guangdong	Shu LI	5	3
Mainland China	Anhui Provincial Hospital	No.17 Lujiang Road 230001Hefei Anhui	Shandong YE	2	1
Mainland China	Shanghai Huashan Hospital, Affiliated to Fudan University	No.12 Wulumuqi Zhong Road 200040 Shanghai Shanghai	Yiming LI	4	4
Mainland China	Zhu Xianyi Memorial Hospital of Tianjin Medical University	6 huanrui North Road, Beichen District 300070 Tianjin Tianjin	Liming Chen	7	5
Switzerland	Luzerner Kantonsspital	Endokrinologie / Diabetologie 6000 Luzern 16	Stefan Fischli	1	1
Switzerland	Diabetes Adipositas Zentrum Zürich	Rietholzstrasse 4 8125 Zollikerberg	Birgit Bach-Kliegel	8	7
Switzerland	Kantonsspital Olten	Stoffwechselzentrum Baslerstrasse 150 Haus M/Eingang Ost 4600 Olten	Thomas Zueger	5	5
Canada	Recherche Clinique Sigma inc	14008 boul Henri-Bourassa Suite 210 G1G 3Y8	Guy Chouinard	4	2
Canada	Diex Recherche Quebec Inc. Plc de la Cit 2600 Blv Laurier Suite 293	Plc de la Cit 2600 Blv Laurier Suite 293 G1V 4T3 Quebec	Andre Frechette	2	0

	G1V 4T3<br>Quebec				
Canada	Recherche GCP Research	6455 Beaubien East<br>2nd Floor<br>H1M 1B1	Giuseppe Mazza	3	2
Canada	Wharton Med Clin Trials	Ste M14<br>414 Victoria Ave N.<br>L8L 5G8 Hamilton Ontario	Sean Wharton	8	6
Canada	Medical Trust Clinics, Inc.	247 Simcoe Street N<br>Suite 101<br>L1G4T3 Oshawa	Hamilton Jeyaraj	3	2
Canada	LMC (Thornhill)	1600 Steeles Avenue West<br>Unit 5-10<br>L4K 4M2 Concord	Robert Schlosser	10	8
Canada	Western Univ. Cnt for Studies in Fam Med	PHFM Bldg<br>1465 Richmond St, Room 2012<br>N6G 2M1 London	Stewart Harris	7	5
Canada	Bluewater Clin Res Group,Inc	481 London Road<br>N7T 4X3 Sarnia	Sean Peterson	6	6
Canada	LMC Clin Rsrch Inc. (Montreal)	6363 Transcanadienne<br>Ste 238<br>H4T 1Z9 Saint Laurent	Elie Sahyouni	4	3
Canada	LMC Endo Centres Ltd.(Bayview)	1929 Bayview Avenue<br>Suite 107<br>M4G 3E8 Toronto	Oren Steen	3	1
Brazil	Centro de Diabetes Curitiba	Rua Alcides Munhoz, 433<br>4º andar, sala 448<br>Bairro Mercês 80810 040	André Vianna	15	11
Brazil	BR Trials – Ensaios Clínicos e Consultoria Ltda.	Praça To+B122mas Morus, 81, 9º andar<br>Agua Branca<br>São Paulo - SP 05003 090	Bruno Halpern	12	10
Brazil	Instituto São José dos Campos em Pesquisas Médicas	Rua Santa Luzia, 56<br>Vila Ema	Marcio Pereira	15	15
Brazil	Quanta Diagnóstico Nuclear	Medicina Nuclear Alto da XV Rua Almirante Tamandaré, 1000<br>80045-170 Curitiba	Rosangela Rea	11	10

Brazil	Instituto de Ciências Farmacêuticas de Estudos e Pesquisas	Avenida Rio Verde, Qd 06 Lotes 01/07<br>Cidade Vera Cruz<br>74935-530	Sergio Vencio	14	10
Brazil	Centro de Diabetes Metabolismo e Endocrinologia	Av. Santos Dumont, 5753<br>Sala 1110<br>Complexo São Mateus – Torre Saúde<br>Bairro<br>Papicu	Adriana Forti	8	7
Hungary	MED-TIMA Kft.	Belvárosi Anyagcserecentrum<br>Gyöngyház u.2 1/4.<br>1132 Budapest	Tímea Tanczer	6	5
Hungary	PTE-AOK II. Belgyogyaszati Klinika es Nephrologiai Centrum	utca 1.<br>7623 Pécs<br>Baranya Vármegye	Istvan Wittman	4	3
Hungary	Debreceni Egyetem	Belgyógyászati Klinika<br>Nagyerdei körút 98.<br>4032 Debrecen<br>Hajdu-Bihar	Éva Katona	29	19
Hungary	Debreceni Egyetem, Kenézy Gyula Kórház	Klinikai Központ<br>Belgyógyászati Klinika<br>D épület<br>Bartók B. u. 2-26.<br>Belgyogyaszat, 2. em.<br>4043 Debrecen<br>Hajdu-Bihar	Gizella Petro	7	7
